# The importance of in-country African instructors in International experiential training programs; a qualitative case study from the university of Minnesota

**DOI:** 10.1186/s12909-023-04129-z

**Published:** 2023-03-03

**Authors:** Faith Nawagi, Aruna Raman

**Affiliations:** 1 School of Medicine, Makerere University College of Health Sciences, Kampala, Uganda; 2 University of Minnesota, Minneapolis, USA

**Keywords:** African instructors, Virtual mobility, Global Health, International Experiential training programs

## Abstract

**Background:**

For effective delivery of international experiential training programs, many universities in the global north, have created partnerships with universities in the global south especially in Africa to enhance their capacity and diversity of learning for their students. However, there is hardly any literature that exhibits the importance of African instructors in international experiential learning programs. This study aimed at establishing the importance of African instructors in international experiential learning programs.

**Methods:**

This was a qualitative case study that examined the importance of instructors and experts from Africa in influencing student learning processes and outcomes in the GCC 3003/5003 - Seeking Solutions to Global Health Issues. Semi-structured interviews with (2) students, (2) University of Minnesota lead faculty for the course, and (3) in-country instructors/experts from countries in East Africa and the Horn of Africa were conducted. Data was analyzed thematically.

**Results:**

Four themes were identified: (1) Filling gaps in knowledge, (2) Orchestrating partnerships for practical exposure, (3) Improving the quality of training, and (4) orchestrating professional personal growth for students. The African in-country course instructors/experts contributed to student learning by providing a true picture reflection of happenings on the ground.

**Conclusion:**

The importance of in-country African instructors’ can be viewed as that aimed at validating students’ ideas to apply to the local settings, streamlining students’ focus, providing a platform for multi-stakeholder engagement to a particular topic, coupled with bringing an in-country context experience in the classroom.

## Introduction

The growing demand for internationalization in higher education has led to the transformation of higher education policies over the past decades [1]. According to the Organization for Economic Co-operation and Development (OECD), internationalization in higher education refers to the integration of international/ intercultural dimensions into all activities of a training institution, i.e., teaching, curricular, staff, and structural function [2]. Various institutions have adopted internationalization by designing international experiential learning programs which include internships, research, study abroad, global health courses, and incubation hubs among others [3]. Experiential learning is the process through which students develop knowledge, skills, and values from direct experiences outside their traditional academic setting [4]. These programs create opportunities for academic inquiry thus promoting interdisciplinary learning, civic engagement, career development, cultural awareness, leadership, and other professional and intellectual skills [5]. For learning to be considered experiential, it must foster creativity, reflection, critical analysis, and opportunities for students to take initiative, make decisions and be accountable for the results among others [4]. For the effective delivery of these programs among many universities in the global north, partnerships have been created with universities in the global south to enhance their capacity and diversity of learning for students at these northern institutions [6]. However, there is hardly any literature that profiles the importance of the global south experts/ partnerships in aiding the learning of the northern students in such programs [7].

The Open Doors program supported by the US Department of State’s Bureau of Education and Cultural Affairs exhibits a profile of the majority of the US universities that have adopted international experiential learning programs to foster transformative learning [8]. Among these is the University of Minnesota which offers a variety of international experiential learning courses [9]. Some of these courses at the University of Minnesota have actively involved in-country instructors and experts from Africa to support students learning. This has been an important approach that has seen remarkable value addition to the University of Minnesota’s international experiential learning courses with realistic transformative and innovative experiences for the trainees. Exhibiting the importance of African instructors in the global north experiential learning programs justifies how African real-in-country experiences can be enhanced and brought into the classroom for the global north students. Furthermore, showcasing the importance of African instructors in international experiential learning programs also exhibits how the global south also contributes to the academic transformative learning of the global north students. While the University of Minnesota and some universities in the United States have actively involved in-country instructors and experts from Africa in their international experiential learning programs, there are hardly any international experiential learning programs that describe the importance, process, and approaches used to ensure effective training and skill gain in partnership with the global south [10]. To be specific, there are hardly any papers that describe the importance of African instructors in international experiential learning programs[7]. This paper seeks to address this gap by establishing the importance of in-country African Instructors in international experiential learning programs offered by the University of Minnesota. The null hypothesis used in this study was, “African in-country instructors and experts are not important in international experiential learning programs offered by the University of Minnesota.”

## Methodology

### Study design

A case study design using a qualitative approach was used to collect data from the in-country instructors and experts from Africa, course faculty, and students enrolled in the program focusing on Africa. Semi-Structured interviews were used to collect data from the participants. All study procedures adhered to the various research guidelines and regulations.

### Study context and setting

The experiential learning programs that involve partnerships and roles with experts in the Global South at the University of Minnesota are under the Grand Challenge Curriculum (GCC) [9]. These are two and include; GCC 3005/5005 - *Global Venture Design: What Impact Will You Make?* and GCC3003/5003 - Seeking Solutions to Global Health Issues) [9]. GCC courses are hosted by the University of Minnesota’s Provost’s office and are available to students across the University. This brings a diverse set of student interests to the courses. The specific courses of interest encourage students to address various challenges locally in Minnesota and internationally especially in mid to low-income countries [9]. These employ various learning approaches such as classroom lectures, group discussions, assignments, and group project development. The courses are open to students at the graduate and upper-undergraduate levels from any discipline. GCC course faculty at the University of Minnesota design the course curriculum, and assessment tools, draw an activity map for the courses, and guide the students through various classroom teachings, group discussions/ presentations, and assignments.

The GCC 3005/5005 aims at having students work in interdisciplinary teams to develop entrepreneurial responses to current health, social and environmental problems while developing the tools, mindset, and skills that can enable them to become leaders in addressing any complex grand challenge [9]. The GCC 3003/5003 course aims to have students work in teams to examine the fundamental challenges to addressing complex global health problems in East Africa and East African Refugee communities in the Twin Cities and develop practical solutions that take culture, equity, and sustainability into account [9]. All activities are aimed at ensuring students have an understanding of the human-centered design to address various challenges, have an innovative mindset, and learn how to work and develop multidisciplinary teams [11]. Furthermore, both these courses ensure students gain skills sought by employers such as being entrepreneurial, analytical, leadership skills, public speaking, and innovation among others [11].

The instruction team for both courses includes in-country partners from India, Somalia, Nicaragua, and Uganda, who develop the various group topics and validate them before offering the courses. After the introductory classroom lectures, the faculty link the student groups to the in-country instructors. The students work and study in groups depending on the challenge and country they choose to focus on. Most importantly, the students have virtual weekly sessions with the in-country instructors which are guided with weekly targets and topics to enable effective student learning.

### Study population

There were 7 participants in this study. These included two students, two University of Minnesota lead faculty for the course, and three in-country instructors/experts from countries in East Africa and the Horn of Africa. Though the interviews comprised in-country instructors and experts in Africa, there is a distinction. In-country instructors work directly with the program, while experts work with a second organization, and provide expertise to the program. The terms might be used interchangeably or in conjunction, throughout this section. There is also clear role demarcation between the course faculty leads and in-country African course instructors. While the former provides theoretical and pedagogical framings, the latter are important to establishing the local context.

Students who were participating in the course and focused on Africa were included. Among these students, those who did not consent were excluded. However, the faculty and in-country African experts and instructors were the only available members of the team that consented. The eligibility criteria were facilitating learning in the GCC 3003/5003 course, having time to participate in the interview, and willingness to provide informed consent. With the faculty and instructors from Africa, in addition to their roles in the course, two of the three African instructors had experience studying and/or working on projects and training institutions in developed countries. Another in-country African instructor had extensive experience working with research and practitioner teams in developed countries and has traveled there frequently. Furthermore, the African in-country experts had rich experience working in the corporate, nonprofit, and international aid environments, and have been part of university environments. They were able to harness their professional networks to bring a richness of expertise to the course.

### Sampling method used

Purposive sampling was used to identify study participants. This was used because of the nature of the study being qualitative and requiring us to get the best-fit participants to gain a deeper insight into the study objectives. Each of the eligible participants was contacted via email for their interest to participate in the study.

### Study tools

The question guide used to interview the participants was developed by the study team. This was reviewed for appropriateness and ability to answer the study objectives. This was a semi-structured interview guide with five questions maximum. These questions sought to establish the views of the participant on the importance of the in-country African instructors and experts in influencing student learning in the GCC 3003/5003 course. These were semi-structured including closed and open-ended questions that allowed in-depth descriptions of participants’ views. The questions assessed the participant’s views on having in-country instructors as part of the training and how their role was important.

### Data collection

The interviews were conducted face to face with the case of University of Minnesota course faculty, via email with the case of students, on Skype for two of Africa’s in-country experts, and via email with a third African in-country expert. Participants were approached via email, thereafter, consent forms and interview guides were emailed to the participants at least 2 days before the interview to enable substantive preparation. We conducted four interviews face-to-face and via skype in total each lasting an hour. Recordings were done for face-to-face and virtual interviews. For email interviews, (3) each of the participants was given two weeks within which to respond. The study recruited all the faculty, in-country African instructors, and experts as well as the students that were participating in the course. We, therefore, did not have a point of saturation however, the strength was all those eligible were included. Characteristics like international exposure, working experience in international experiential learning programs, gender, age, and country location were captured from the participants.

### Data analysis

Thematic analysis was employed, and this was done by manually reviewing the transcripts several times to identify meaningful units and texts to develop codes using open coding (generation of initial concepts from the data collected). Through axial coding, the codes generated from the transcripts were categorized from which emerging subthemes and themes were generated.

### Quality control

The trustworthiness and rigor of this study given its qualitative nature were observed using Lincoln and Guba’s (1985) trustworthiness criteria and techniques [12]. For credibility, prolonged engagement of the participants, having the research team review the findings, and review by a qualitative Analysis expert was done. To enhance credibility, we had to identify our biases to ensure they do not interfere with the findings by applying reflexivity during data collection and analysis. We did this by recording each interview and immediately transcribing after the interview, taking notes during the interview from the participants and our thoughts, and continually developing and editing our subjective statements. The results were shared with the participants especially the faculty at the University of Minnesota to ensure the findings are aligned with the data collected. Furthermore, the collection of data from different participants using three different approaches were done to ensure triangulation. A detailed description of the qualitative data collection and analysis process was done to ensure transferability in similar contexts elsewhere. To observe the dependability of the findings, thematic analysis and review by a qualitative data expert were done to derive findings. To observe confirmability the study findings were reviewed by the study team for accuracy and alignment with the study objectives.

## Results

Four themes to reflect the importance of in-country instructors and experts were obtained as shown below.

### Filling gaps in knowledge

The in-country African instructors/experts are the “feet on the ground” - they provide information on realistic and relevant contexts since the students are unable to visit the countries during the course. The experts appreciate the uniqueness of the course structure in allocating a prominent place for expertise from Africa. This is exhibited in the quotes below.

*We leverage our knowledge to guide students to examine various problem statements and existing approaches and apply their learning from the course to the formulation of solutions that are creative, sustainable, and apply to the local context. As we do this, we are cognizant of our own bias and how that can influence student learning. I pay attention to my own intrinsic bias and ensure that I point the students to existing literature to validate the concepts at hand (African Instructor Interview, November 2019)* [13].

*In-country African instructors are important in validating secondary information, obtained through online research. Even the most respected peer-reviewed journal articles might fail to capture cultural nuances (student email interview, November 2019)*[13].

### Orchestrate meaningful partnerships for practical exposure

The African instructors are instrumental in establishing interaction processes and meeting schedules with the students. They emphasized that this consistency in interactions is important in the development of ideas, and the continuity of the learning process. Each week, the team discusses new ideas. The African in-country instructor vets each idea and suggests pathways for further exploration. Where the need arises, the primary in-country African instructor connects students to other experts, through a careful introduction process. This is shown in the quote below.

*I personally meet other experts, especially from organizations doing work around a similar concept the students are studying. I tell them about the students’ focus. Through (Skype) calls, I introduce both the expert and the student team. I tell them about moving forward, (and) validate the ideas. I do the groundwork to see that they are on the same page before they meet. (Personal interview, November 2019)* [13].

### Improvement of the quality of training

The course faculty view the in-country African instructors as being instrumental in providing practical information, enabling enhanced discussions, and having enabled improved quality of projects developed by the students as shown in the quotes below.

*They (African instructors and experts) strengthen my teaching (personal interview, November 2019)*[13].

*We have always had superstar students - you can do anything with them. (However), the quality of good work has also become consistent since we started to work with the African instructors and experts .” (Faculty instructor personal interview, November 2019)* [13].

### Orchestrate professional personal growth among students

Students view interactions with global south instructors and experts as part of their personality growth, especially in the domain of leadership, confidence, cross-cultural interaction, and communication skills. This is exhibited in the quote below.

*Through navigating cross-cultural interactions with the African instructor, I have learned to reach out to contacts, ask questions in meetings, raise concerns, make suggestions, summarize ideas into messages, and more (Student email interview, November 2019)* [13].

*I recognize the role in developing cultural respect, and a broader, more diverse worldview. (Student email interview, 2019)* [13].

*It has given me leadership skills, improved communication skills, bravery, a more open mind, and a stronger work ethic (Student email interview, November 2019)* [13].

## Discussion

Having in-country African instructors/ experts for the international experiential learning programs at the University of Minnesota has seen a remarkable value addition to the program. Students have had a realistic transformative experience [10] given the ability to ensure validation of ideas, creative innovation applicable to the varying settings with a sensitivity of appropriateness, existent technology, addressing bias, and realistically understanding the factors around a given challenge from a firsthand person living in these countries.

This approach evidences collaborative global learning [14] where the faculty, in-country instructors from Africa, and students work together to construct knowledge which then enables students to combine perspectives and theories thus enabling them to gain global innovation competencies required and applicable in their careers. This kind of learning and partnership is in line with the Sustainable Development Goals (SDGs) specifically, SDG17 which promotes the triangulation of partnerships between developing and developed countries for the effective achievement of the set goals by 2030 [15]. Although there is much literature about how developing countries learn from developed countries and economies [16], this paper adds to the body of knowledge exhibiting how developed countries are learning from the developing countries’ systems, approaches, and innovations. This indeed provides an opportune platform to leverage learning from the African context, system, and approaches for the students.

Despite the widely documented hardships in virtual learning platforms, and internet connection in Sub-Saharan Africa [17], this program evidences the progressive growth in internet connection, virtual communication medium, information technology, and literacy in Africa. The students could set up an opportune flexible time to meet with instructors weekly through various communication platforms like WhatsApp and skype which are often clear, in addition to timely email communications. This is further evidenced by the World Bank which affirms an improvement in internet speed, Information Communication and Technology (ICT) skills, literacy, and use in the region [18]. The virtual meetings with the in-country African instructors further evidence the reliability of the virtual medium as an effective mode in promoting learning in a very cost-effective way for the students compared to if students had to travel to these countries. It provides a platform to learn multi-stakeholder engagement, understand the existing systems, and how to interact with them to ensure the effective implementation of a program.

While having in-country African instructors has played a big role in enhancing learning in the GCC 3003/5003 - Seeking Solutions to Global Health Issues course, it is important to note that bias from both the faculty and the African instructors is likely to influence students’ learning. However, as mentioned by one of the African instructors, they are able to be aware of their own bias and ensure appropriate scientific material is provided to the learners to enable them to arrive at a conclusion.

The small size of the study could be a limitation; however, these were the only integral people available since they are the only in-country African experts and faculty the program works with and the students who volunteered to participate. Furthermore, one of the instructors has worked with the program for over five years, and the other two experts represent a reputable nonprofit, which enables a wealth of experience and well-thought-out judgment of roles. Despite being small, the study has used a qualitative approach that allows in-depth discussions and involved three in-country African instructors from two East African countries, two lead faculty with a multitude of global experience, and two students, thus a diverse view and realistic analysis of the intended objectives. The purpose of keeping this as a small pilot study is to enable the refinement of design and methodology, so future studies benefit from the learning in this field.

While this pilot study establishes the importance of African in-country instructors and experts in international experiential learning programs, there is a need for a more systematic analysis, involving various institutions, and a broader examination of African/Global-South expert roles in influencing the learning process and outcomes in international experiential learning programs in Universities in the USA and the wider Global-North. Furthermore, studies that detail the role of African instructors in curriculum development for international experiential learning programs are key.

## Conclusion

In-country African instructors’ importance can be viewed as that aimed at validating students’ ideas to apply to the local settings, streamlining student’s focus, and providing a platform for multi-stakeholder engagement to a particular topic coupled with bringing a real country picture experience and context in the classroom from a different country. They provide a conduit and platform for students to learn various approaches to communication and knowledge acquisition from a different context but are key in guiding their interventions.

## Data Availability

Data generated has been shared in this manuscript as texts in the results section. Furthermore, data tools are available upon request from both authors.
